# Spatial Scaling of Non-Native Fish Richness across the United States

**DOI:** 10.1371/journal.pone.0097727

**Published:** 2014-05-20

**Authors:** Qinfeng Guo, Julian D. Olden

**Affiliations:** 1 USDA FS, Eastern Forest Environmental Threat Assessment Center, Asheville, North Carolina, United States of America; 2 School of Aquatic and Fishery Sciences, University of Washington, Seattle, Washington, United States of America; University of Windsor, Canada

## Abstract

A major goal and challenge of invasion ecology is to describe and interpret spatial and temporal patterns of species invasions. Here, we examined fish invasion patterns at four spatially structured and hierarchically nested scales across the contiguous United States (i.e., from large to small: region, basin, watershed, and sub-watershed). All spatial relationships in both richness and fraction between species groups (e.g., natives vs. exotics) were positive at large scales. However, contrary to predictions using null/neutral models, the patterns at small scales were hump-shaped (unimodal), not simply negative. The fractions of both domestic (introduced among watersheds within the USA) and foreign (introduced from abroad) exotics increased with area across scales but decreased within each scale. The foreign exotics exhibited the highest dominance (lowest evenness) and spatial variation in distribution, followed by domestic exotics and natives, although on average natives still occupy larger areas than domestic and foreign exotics. The results provide new insight into patterns and mechanisms of fish species invasions at multiple spatial scales in the United States.

## Introduction

Understanding spatiotemporal patterns and drivers of species invasion remains a central challenge in ecology. Among the heavily debated issues in invasion ecology is the spatial scaling of native-exotic richness relationships and exotic fraction (a measure of “degree of invasion” or DI) within and across locations. Three main factors contribute to the highly variable nature of this relationship: (1) data quality (e.g., whether domestic species are accounted for), (2) spatial scale at which this relationship is investigated, and (3) metrics used to assess habitat invasibility or DI [1], [2], [3], [4], [5]. In addition, the intensity, extent, and accuracy of data related to species invasions are remarkably inconsistent among, and even within, regions and taxonomic groups, leading to additional uncertainty in native-exotic richness relationships.

Elucidating similarities and differences in native-exotic richness associations across scales, regions, and taxonomical groups would offer critical and timely information for both basic and applied research [6], [7]. There exists a rich history of research on fish invasion and homogenization at biogeographical scales [8], [9], [10], [11], [12], [13]. However, little attention has been given to investigations across multiple spatial scales. Recent decades have witnessed considerable advancements in both the quantity and quality of data pertaining to the intentional introduction of exotic fish species, thus providing new opportunities to tackle the critical scaling issue associated with native-exotic richness associations (scaling-up and -down) that have continued to challenge ecologists.

Multiple but not mutually exclusive mechanisms and null/neutral models have been proposed to explain the diverse patterns related to native vs. exotic diversity observed at different scales and for various organisms [2], [3], [14], [15], [16], [17]. However, given the large differences among organisms in many aspects (size/dispersal), different taxonomic groups are likely to show different patterns at various spatial scales (extent and grain). Without field survey/observations, existing theories are challenged to differentiate the spatial scales at which positive or negative native-exotic richness relationships may manifest. This is particularly evident when species introductions are unevenly distributed across space and time. Given the relative nature of scale (large vs. small) and diversity of taxonomic group (e.g., fishes vs. plants in terms of species richness), knowledge regarding when and how native-exotic relationships change over space and time is needed.

Here, we examine patterns of richness and distribution for native and exotic (both foreign and native translocations) fishes across four nested scales of the contiguous United States. We address: (1) how degree of invasion by exotic fishes varies among and within spatial scales as defined by nested watershed levels, and (2) what are the main drivers for the observed patterns across scales. We also use native species across watersheds as a background reference for comparison to describe and interpret spatial patterns of exotic species invasion [18]. The answers to these questions would facilitate our understanding of fish invasion and homogenization and advance the scientific knowledge in freshwater conservation biogeography [19].

## Methods

We collated native and non-native species occurrence for watersheds of the contiguous United States using the NatureServe [20] and USGS NAS (2012; http://nas.er.usgs.gov/) databases, respectively. Fish occurrences were based on hierarchically and spatially nested watersheds across the contiguous United States. For this study, the nested watersheds were classified based on the Watershed Boundary Dataset into four levels (spatial scales) according to the Hydrologic Unit Codes (HUCs): 18 hydrologic regions (2-digit HUC; the largest), 204 river basins (4-digit HUC), 333 watersheds (6-digit HUC), and 2073 sub-watersheds (8-digit HUC; the smallest). The database included a total of 935 fish species consisting of 774 native species (of which 295 were “*domestic exotics*” introduced to at least one watershed) and 161 “*foreign exotics*” that origin from outside the United States. Importantly, nativity status of a species depends on the spatial grain of investigation. For example, a fish species might be native at the largest scale (2-digit HUC) but could be either native or non-native (domestic exotic) at smaller scales (4-, 6-, and 8-digit HUCs).

We determined native and non-native species occurrence for sub-watersheds of the contiguous United States using the NatureServe Central Database [20] and the United States Geological Society (USGS) Non-indigenous Aquatic Species Database (2012), respectively (Fig. S1. The NatureServe Central Database contains primarily native species occurrences that were derived from state natural heritage programs, the scientific literature and from species experts. The USGS Non-indigenous Aquatic Species Database contains non-native species occurrences (defined as a species introduced from outside its native range) that were sourced from the literature, museums, databases, monitoring programs, state and federal agencies, professional communications, online reporting forms, and Aquatic Nuisance Species hotline reports. Together, these two databases represent the most complete distributional information possible for freshwater fish species across the United States and provided the basis for regional freshwater conservation assessments [21].

We recognize two general drawbacks associated with of the use of biodiversity databases: (1) lack of data on survey effort, and (2) incomplete coverage of the geographical and environmental diversity that affects the distribution of the organisms [22]). Both of these concerns are well recognized to limit the inferences drawn from large-scale analyses [23]. Although data on survey effort is not available (and thus represents a data limitation), the diverse data sources ensure a comprehensive spatial coverage of freshwater ecosystems across the United States (described above). For example, non-species occurrences were sourced extensively from national biomonitoring efforts that involved systematic, probability-based surveys or were synoptic in scope and sampled locations from a broad range of stream types and sizes across the United States.

The exotic fraction – area relationships were examined using regression analysis and comparisons among spatial scales were conducted using analyses of variance (ANOVA). The comparisons between domestic and foreign exotic fractions were made for each of the four individual watershed scales using paired Student *t* – tests. We comparatively examined the relationships among variables (or measures) such as species richness, exotic fraction (a surrogate for measuring degree of invasion or DI), domestic and foreign exotics, and area, across the four spatial scales and within each scale when appropriate. We first used simple regression analyses to test whether a relationship existed between the paired variables. When such analysis failed to detect any relationship, we deployed quantile regressions (both first and second order) to test possible relationships [24], [25]. Quantile regression estimate rates of change in all parts of the distribution of a response variable, which is well suited to examine relationships between native and exotic species richness in cases where other influencing factors are unmeasured and unaccounted for, as is often the case [9]. Rather than focusing exclusively on changes in the mean response that can lead to underestimation, overestimation, or a failure to detect changes in heterogeneous distributions [25], quantile regression estimates multiple rates of change (slopes) in responses with unequal variation. The quantile regression analyses were conducted for six quantiles (0.1, 0.25, 0.5, 0.75, 0.9, and 0.95) using PROC QUANTREG in SAS [26] for the sub-watersheds where data were most appropriate for such analysis [25].

## Results

**Figure 1:**
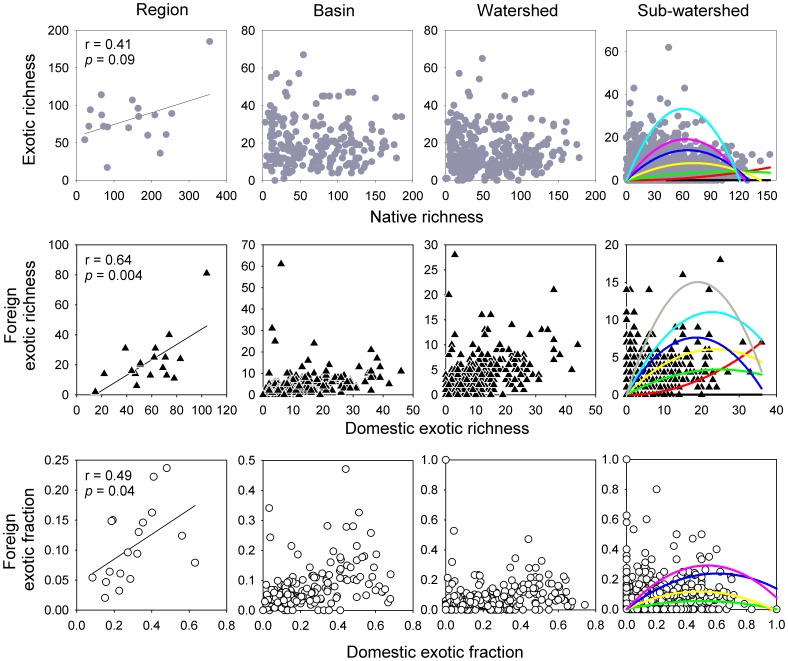
Relationships between native and exotic richness (top), domestic and foreign exotic richness (middle), and domestic and foreign fraction (bottom) across multiple spatial scales in the contiguous United States. In each panel for sub-watersheds, the second order regression curves were based on quantiles of 0.1, 0.25, 0.5, 0.75, 0.9, and 0.95 from bottom to top, respectively; and the corresponding equations and significance were given in Table 1. Exotic richness included both foreign and domestic exotics.

At each of the four spatial scales, native fishes had the highest richness, followed by domestic exotics and foreign exotics (Fig. S2). All the spatial relationships examined here in both richness and fraction between species groups switched from positive (linear regression) at the larger hydrologic region scale to unimodal (quantile regression) at smaller scales where the patterns became more complicated (Fig. 1). For example, for higher quantiles (i.e., 0.9, 0.95) at the sub-watershed level, the native-exotic, domestic-foreign exotic relationships were unimodal (or hump-shaped) better fitted with second-order regressions. In other words, when native richness was low, its relationship with exotic richness was positive; but as native richness continued to increase, their relationships became negative. By contrast, for lower quantiles (i.e., 0.1, 0.25) the relationships were mostly linear and positive. The same was true for the domestic-foreign exotic relationships at the sub-watershed level regardless if exotic richness or fraction was used (Figs. 1, S3; Table 1). Quantile regression on watersheds at basin- and watershed-levels yielded very similar results but with less statistical support (not shown).

**Table 1:** Second-order quantile regression relationships between paired variables related to native and exotic species based on data from sub-watersheds throughout the contiguous United States (*n* = 2073) (see also Fig. 1 for graphical illustrations).

Variables	Model equations [*y = f(x)*]	Quantile	*P*
Native (*x*) – exotic richness (*y*)	– *x*+*x* ^2^	0.1	<0.0001
	0.0011–0.0013*x* +0.0003*x* ^2^	0.25	<0.0001
	0.9693+0.0307*x*+*x* ^2^	0.5	0.85
	4.593+0.0267*x*+*x* ^2^	0.75	0.89
	8.19+0.0777*x* –0.0004*x* ^2^	0.9	0.27
	1.81+0.1009*x* –0.0007*x* ^2^	0.95	0.21
Domestic (*x*) – foreign exotics (*y*)	– *x*+*x* ^2^	0.1	<0.0001
	–0.0056*x* +0.0056*x* ^2^	0.25	<0.0001
	0.27*x* –0.0055*x* ^2^	0.5	<0.0001
	0.64+0.36*x* –0.0051*x* ^2^	0.75	0.0008
	2+0.34*x* –0.0041*x* ^2^	0.9	0.04
	3+0.24*x* +0.006*x* ^2^	0.95	0.14
Domestic (*x*) - foreign exotic fraction (*y*)	*x* – *x* ^2^	0.1	<0.0001
	– *x*+*x* ^2^	0.25	<0.0001
	0.22*x* –0.22*x* ^2^	0.5	<0.0001
	0.01+0.39*x* –0.38*x* ^2^	0.75	<0.0001
	0.04+0.5*x* –0.31*x* ^2^	0.9	<0.0001
	0.07+0.63*x* –0.46*x* ^2^	0.95	0.004

**Figure 2:**
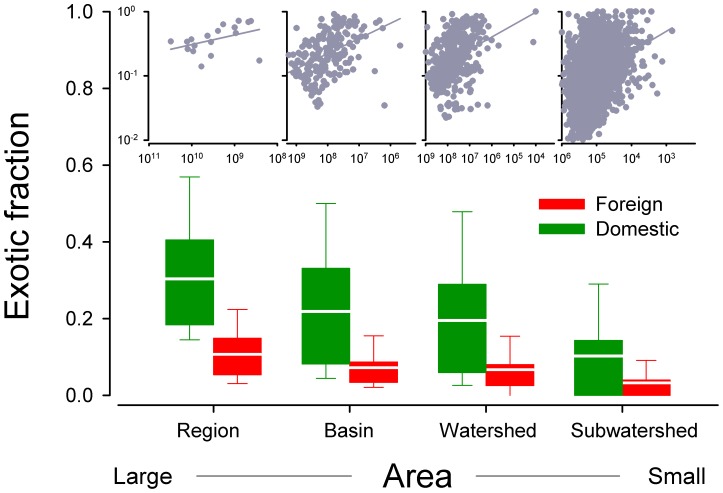
Relationship between the fractions of both foreign and domestic exotic fish and surface area across multiple spatial scales. Foreign exotic fish accounted for a much lower overall exotic proportion than domestic exotic fish (The horizontal bars represent means across the four spatial scales). Note one sub-watershed from Florida with extremely high exotic fish richness is regarded as an outlier and then removed from the analysis.

Across the hierarchy of spatial scales represented by nested watersheds, the fractions of both domestic and foreign exotics, as a measure of degree of invasion (DI), increased with area (ANOVA, domestic fraction: *F* = 74.08, *df = *3, *P*<0.0001; Foreign fraction: *F* = 43.23, *df* = 3, *P*<0.0001). Also, domestic exotic fraction was significantly higher than foreign exotic fractions across the four spatial scales (i.e., regions, basins, watersheds, and sub-watersheds; paired *t* - test, *T* = 5.16, *P* = 0.014) and within each scale (in all paired *t* – tests for each of the four scales, *P*<0.0001; Fig. 2). However, within each of the four scales, the reverse pattern was observed; i.e., the exotic fraction decreased with watershed area (Fig. 2 insert). This was true when domestic and foreign exotics were analyzed separately or in combination (results not shown).

## Discussion

### Native-exotic Relationships Vary Across Scales

**Figure 3:**
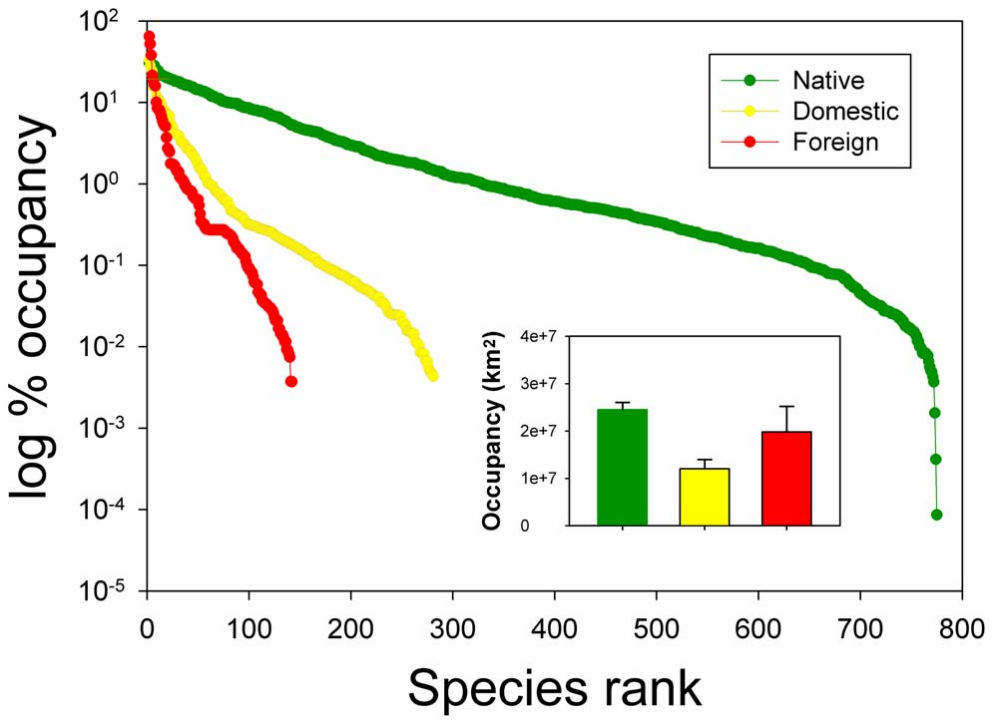
Species rank curves for the three groups (natives, domestic exotics, and foreign exotics) using the distribution data at sub-watersheds level in the contiguous United States. Although foreign exotics still had smaller distribution than natives (but higher than domestic exotics; see inserted panel [mean ± SE]), the variation among species was much larger which was in agreement with the occupancy curves of the three species groups.

Consistent with field observations and theoretical predictions, all spatial relationships in both richness and fraction between species groups (e.g., natives vs. exotics, foreign vs. domestic exotics) are positive at large spatial scales. However, in direct contrast with the simple negative relationships predicted by null/neutral models, the small-scale patterns may be better described by either unimodal relationships or constraint envelopes. Positive relationships at larger scales are expected if large spatial extents are associated with more space, habitat types, and resources that are available for both natives and exotic to coexist [2], [15]. It is also possible that, over large scales, null/neutral models may simply explain the positive relationships without considering species interactions or by potentially averaging over sampling biases. However, at such scales, even though competition among species or species groups (natives and exotics) occurs, exotic species may only replace individuals of some native species, not their entire populations [27]. At small scales, the opposite would be mostly true thus competition and/or predation from natives or all resident species (natives plus exotics) becomes more intense thus resisting further invasions [28], [29]. However, at the smallest scale (sub-watershed) in this study, we observed unimodal patterns rather than clear negative relationships, indicating the limitations of null/neutral models in explaining this observation [30]. However, at smaller scales, it is also plausible that humans have introduced species more frequently to highly disturbed areas with high human densities where native species diversity has been substantially reduced [6], [31], leading to a negative relationship (the second half of the unimodal relationship) that may not have much to do with heterogeneity or resource use. The first half of the unimodal curve (positive) could be due to the fact that, in habitats far from saturation by species (i.e., low native richness) yet space and resources are not limited (similar to the situation at large-scales), both native and exotic species can continue to increase (either through speciation/colonization or human introduction). At present, the foreign exotics exhibited the highest dominance (lowest evenness) and spatial variation in distribution, followed by domestic exotics and natives, although on average natives still occupy larger areas than domestic and foreign exotics (Fig. 3). We urge that additional research focuses on how the relative distribution area of natives vs. exotics (both domestic and foreign; Fig. 3) influences native-exotic richness relationships across scales.

It is interesting to note the positive relationship between domestic and foreign exotic fishes and their similar relationships with native species. In agreement with previously proposed mechanisms for plant invasions in the United States, the positive relationships between domestic and foreign exotic richness especially at large scales (i.e., regions) may indicate that either both groups of species have been introduced in the similar ways or they may respond to the same factors that influence colonization success, of which habitat is one of several factors [32]. By contrast, unlike observation in many field research and simulations, we did not observe strong negative native-exotic relationships at the smallest scale (sub-watersheds). There are two possible reasons. First, in contrast to individual streams [30], the sub-watershed level may be too large (e.g., not fully invaded) for the negative relationships to manifest. Second, the strong negative native-exotic relationship may not actually exist for fishes in the United States, even at smaller scales [9]. Nevertheless, the emergence of increasingly negative relationships at the sub-watersheds in which native richness is higher might indicate the presence of stronger competition or even species saturation [2](Fig. 1).

The issue of native-exotic relationships being scale dependent has most frequently discussed for plants [33], [34]. Our study of freshwater fishes suggests that such scale-dependency may also apply to aquatic ecosystems. However, contrary to most claims of simple negative relationships on small-scales in literature, our results demonstrate that the small-scale patterns observed in our study are much more complex than previously thought (but see [30] for an even smaller scale). Furthermore, as mentioned earlier, even null/neutral theory may explain the changes in the direction of native-exotic richness relationships. Information regarding the particular scales at which the direction of this relationship switches is relevant for invasive species management. For instance, both native and exotic species richness might be supported at spatial scales that exceed the switch point (i.e., species coexist), but at smaller scales exotic species may out-compete native species for resources/space leading to population losses [4], [9], [28], [33].

### Opposite Exotic Fraction-area Associations within and among Scales

The strong scale-dependent patterns in the degree of invasion (DI) by exotic fishes across the United States are somewhat expected, but the exactly opposite patterns observed between cross- and within-scales are surprising. The exotic fraction increases with area across the spatially nested scales from region to sub-watersheds but decreases with area within each individual scale (Fig. 2). Interestingly, these observations are very similar to the patterns for the exotic plants in the United States; i.e., when compared between the state and county levels, exotic fraction increases with area (state>county) but when compared either among states or among counties, the exotic fraction decreases with area; Q. Guo unpublished results).

Declines in exotic fraction with area across all four spatial scales is likely given that the exotic fraction inevitably decreases with increasing area as the pool of potential exotic species decreases (Fig. 2 inserted panels on top). However, the interpretation of the opposing cross-scale relationships is more difficult (i.e., exotic fraction increases with area; Fig. 2). The seemingly contradictory patterns may be caused by the relative dominance level or spread (distribution areas) of each of the three species groups (natives vs. domestic exotics vs. foreign exotics) at different spatial scales. It is also possible that, at smaller scales, exotic richness may be similar across HUCs but composition may differ, leading to an increase in exotic fraction with increasing scale. For example, the exotic-area curve is steeper than the native-area curve, which could lead to greater exotic fractions with area [35]; supporting the notion that the large-scale determinants of non-native fish richness are context-dependent [36] and may differ depending on whether species richness or composition are examined [37].

In conclusion, our multi-scale investigation supports the notion that native-exotic and exotic-area relationships vary across and within spatial scales. However, the results also reveal a very different observation particularly from that in theoretical studies [2], [3], [15], [38]; that is, at small-scale, the native-exotic relationships are not simply or consistently negative, but unimodal. Although, at present, native fishes still have larger distribution areas than exotics, this is likely to change as a result of continued human activities leading to habitat modification and climate change [39]. Future investigations on the effects of climate change on the biogeography of native and non-native species remain an urgent research need.

## Supporting Information

Figure S1 **Map of the study region depicting sub-watersheds of the contiguous United States.** Shading represents total (native and non-native) fish species richness.
(TIF)

Figure S2 **Comparison of species richness of natives, domestic exotics, and foreign exotics across the four scales (region, basin, watershed, and sub-watershed) in the contiguous United States.**
(TIF)

Figure S3 **Sample estimates, i.e., the intercepts and slopes of first- and second-order quantile regressions between paired variables for sub-watersheds.** The blue area represents 95% confidence intervals. Top: Native richness – Exotic richness; Middle: Domestic exotic richness - Foreign exotic richness; Bottom: Domestic exotic fraction – Foreign exotic fraction.
(TIF)
