# Supplementary figures and images for: Spatial Scaling of Non-Native Fish Richness across the United States
Source: PLoS One. 2014 May 20;9(5):e97727. doi: 10.1371/journal.pone.0097727 (PMC4028219; doi:10.1371/journal.pone.0097727)

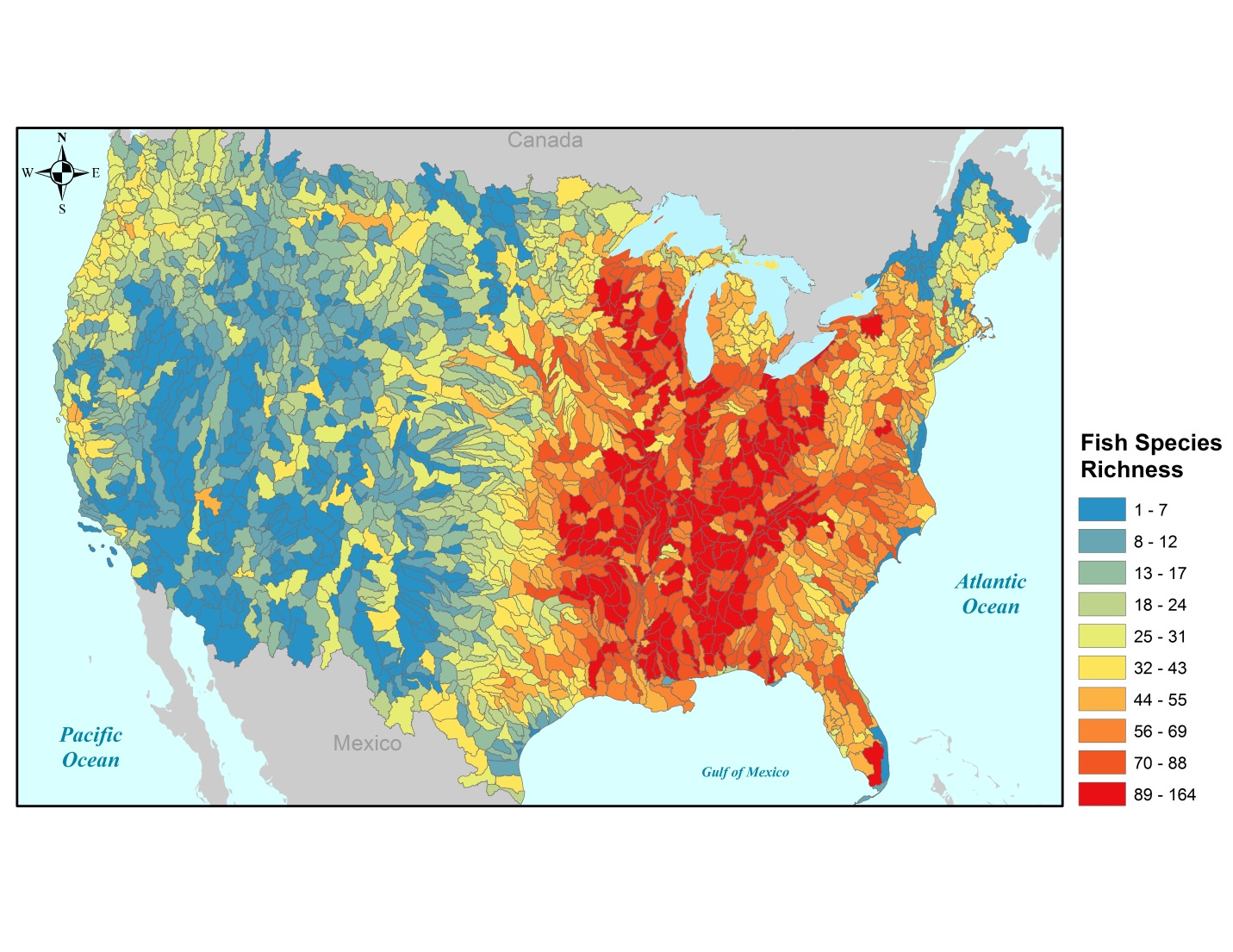

Supplement: Figure S1 — Map of the study region depicting sub-watersheds of the contiguous United States. Shading represents total (native and non-native) fish species richness. (TIF) [file pone.0097727.s001.tif]

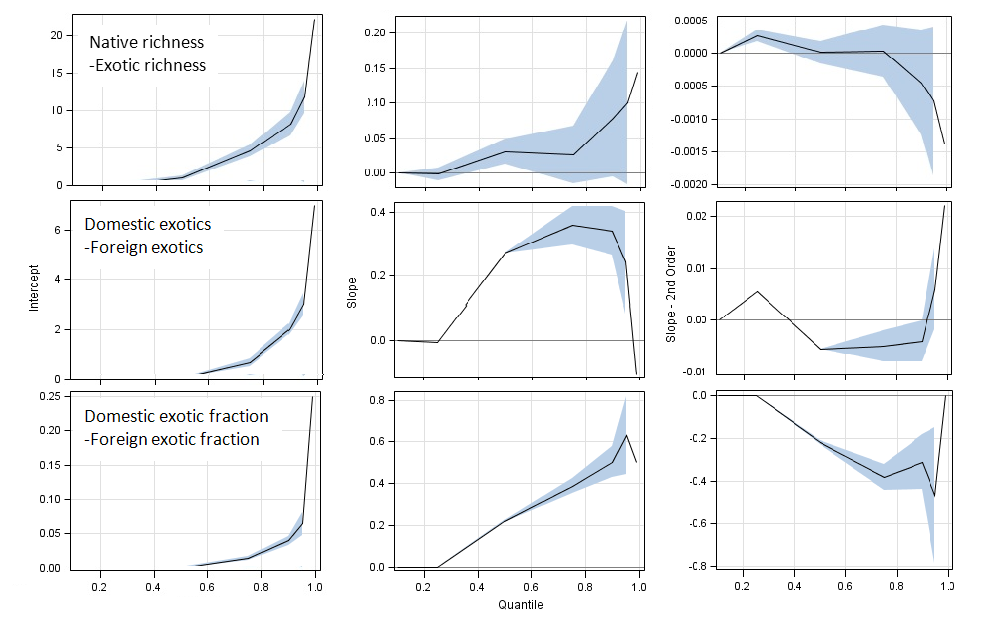

Supplement: Figure S3 — Sample estimates, i.e., the intercepts and slopes of first- and second-order quantile regressions between paired variables for sub-watersheds. The blue area represents 95% confidence intervals. Top: Native richness – Exotic richness; Middle: Domestic exotic richness - Foreign exotic richness; Bottom: Domestic exotic fraction – Foreign exotic fraction. (TIF) [file pone.0097727.s003.tif]
